# Lightness Discrimination Depends More on Bright Rather Than Shaded Regions of Three-Dimensional Objects

**DOI:** 10.1177/2041669519884335

**Published:** 2019-11-22

**Authors:** Matteo Toscani, Matteo Valsecchi

**Affiliations:** Department of Psychology, Giessen University, ‎Hesse, Germany

**Keywords:** lightness/brightness, natural image statistics, object recognition, perception, surfaces/materials

## Abstract

The brighter portions of a shaded complex object are in principle more informative about its lightness and are preferentially fixated during lightness judgments. In this study, we investigate whether preventing this strategy also has measurable detrimental effects on performance. Observers were presented with a reference and a comparison three-dimensional rendered object and had to choose which one was “painted with a lighter gray.” The comparison was rendered with different diffuse reflectance values. We compared precision between three different conditions: full image, 20% of the lightest pixels removed, or 20% of the darkest pixels removed. Removing the bright pixels maximally impaired performance. The results confirm that the strategy of relying on the brightest areas of a complex object in order to estimate lightness is functionally optimal, yielding more precise representations.

## Introduction

The amount of light coming to the eye from a matte object depends on the intensity of the illumination and on the proportion of light that is reflected (i.e., the surface albedo). Albedo is a specific property of the surface’s material and is thus of great importance for material perception. Lightness is defined as the visual counterpart of albedo (Adelson, 2000).

To perceive lightness, the visual system is faced with the problem that it is impossible to distinguish between illumination and reflectance solely based on the light originating from one point of the surface. Different factors contribute to solve this ambiguity. Lateral inhibition between retinal neurons filters out shallow intensity gradients, which are prevalently due to illumination rather than reflectance differences within a surface (Cornsweet, 1970; Land & McCann, 1971; Mach, 1866). Lightness perception also depends on higher order inferences on the scene. The geometrical interpretation of the shape of a surface in its context dramatically changes its lightness (Gilchrist, 1977; Knill & Kersten, 1991; Purves, Shimpi, & Lotto, 1999). The visual system is also able to parse the scene in depth layers and account for the effects of semitransparent media on the reflected light (Anderson, 1997, 2003; Anderson & Winawer, 2005).

As a general theoretical framework, it was proposed that the visual system recovers albedo by explicitly estimating and discounting the contributions of illumination and geometry to luminance (e.g., Marr, 1982; Pizlo, 2001; Poggio & Koch, 1985; Poggio, Torre, & Koch, 1987). This is computationally complex and poses a “chicken and egg” problem, because in order to estimate and discount illumination and geometry, the visual system would need to estimate the surface’s albedo (Fleming, 2014). Alternatively, the system could use simple heuristics to bypass this problem and estimate surface albedo directly from image statistics (see for review: Fleming, 2014; Thompson, Fleming, Creem-Regehr, & Stefanucci, 2011). For instance, it can exploit that albedo is negatively correlated to the skewness of the histogram of the surface luminance distribution and positively correlated to its standard deviation and to its 90th percentile (Motoyoshi, Nishida, Sharan, & Adelson, 2007; Sharan, Li, Motoyoshi, Nishida, & Adelson, 2008).

Our previous research (Toscani, Valsecchi, & Gegenfurtner, 2013a, 2017) indicated that the brightest portions of the luminance histogram are diagnostic for surface albedo. We showed this using a physically based rendering system (Heasly, Cottaris, Lichtman, Xiao, & Brainard, 2014; Ward, 1994) to simulate reflections in a large number of three-dimensional realistic shapes. This approach allows to generate a large dataset of object images to statistically relate reflected light to surface properties (e.g., Singh, Cottaris, Heasly, Brainard, & Burge, 2018; Wiebel, Toscani, & Gegenfurtner, 2015). Specifically, we rendered images of different objects, placed in different complex realistic light fields (Debevec, 1998). The images were rendered with different albedos under different positions and viewing angles. By means of receiver operating characteristic (ROC) analysis (Toscani et al., 2013a) and linear classifiers (Toscani, Valsecchi, et al., 2017), we demonstrated that the highest percentiles of the luminance distributions of matte objects are particularly diagnostic for their albedo. This property is exploited by human observers, who tend to base their lightness judgments on the surfaces’ brightest portions (Toscani et al., 2013a, 2015; Toscani, Valsecchi, & Gegenfurtner, 2013b; Toscani, Valsecchi, et al., 2017; Toscani, Zdravković, & Gegenfurtner, 2016).

When we selectively manipulated different bands of the luminance histograms of three-dimensional simulated objects while keeping their mean luminance constant, we observed that for matte surfaces, increasing or decreasing luminance of the brightest band maximally affected lightness appearance (Toscani, Valsecchi, et al., 2017).

The strategy of using the most diagnostic luminance values as a proxy for lightness appearance would help the visual system to create a perceptual representation which maximally tells surfaces of a different albedo apart, and that is relatively immune to changes in illumination and geometry. In fact, in a complex three-dimensional object, the maximum luminance would be expressed where the local surface is oriented perpendicular to the light source, the rest would be affected by shading and thus depend on the local orientation. This strategy constitutes a possible heuristic to achieve a stable lightness appearance, without requiring knowledge of the scene geometry, shape, or illumination.

While we know that this strategy influences lightness appearance, the direct prediction of our simulations is that lightness discrimination judgments are mostly dependent on the brightest percentiles and was never tested on human perception. In fact, the simulations are based on luminance, but perceived brightness is known to be nonlinearly related to luminance, and, whether this relationship is logarithmic (Fechner, 1860) or roughly a cube-root curve (Stevens, 1957), this implies that differences within the low luminance range might be amplified compared to those in the high range. Hence, at the perceptual level, the dark portion of an object’s luminance distribution might impact discrimination more than the light one, despite being in principle less informative.

Here, we test the relative importance of dark and light portions of the luminance distribution of shaded complex objects in a lightness discrimination task. Removing the lightest portions impaired discrimination more than removing the dark ones.

## Methods

### Participants

Five naive observers took part in the experiment. They all had normal or corrected visual acuity. All gave written informed consent in accordance with the Code of Ethics of the World Medical Association (Declaration of Helsinki) for experiments involving humans. The experiments were approved by the local ethics committee (approval number LEK 2009-0008).

### Apparatus

We used the psychtoolbox-3 software (Kleiner, Brainad, & Pelli, 2007) working on MATLAB (http://www.mathworks.com), to display the rendered movies on Samsung SyncMaster 1100 MB monitor. The procedure for monitor linearization has been described elsewhere (e.g., Ennis, Toscani, & Gegenfurtner, 2017; Milojevic, Ennis, Toscani, & Gegenfurtner, 2018).

### Stimuli

**Figure 1.**
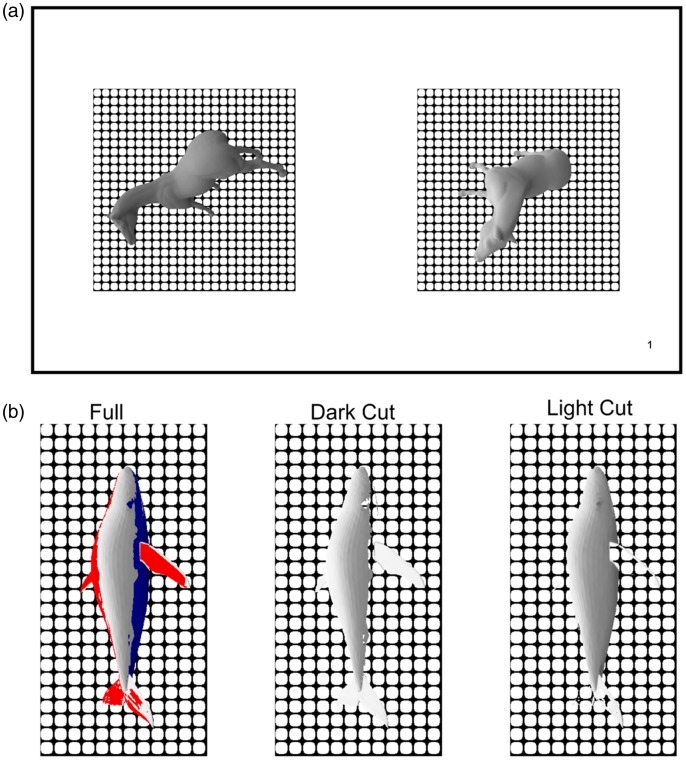
Stimuli. (a) Reference and comparison stimulus, one on the left and the right side of the display. The horse shape is shown here; the other one was the three-dimensional model of a whale; see example in (b). (b) Experimental conditions. In the *Full* condition, the whole shape was presented. Here, for illustration purposes, the 20% lightest and darkest pixels are highlighted in red and blue, respectively. In the *Dark Cut* condition, the 20% dark pixels were substituted with the background pattern; the 20% lightest in the *Light Cut* condition.

We used two three-dimensional models (Figure 1) to render realistic matte objects under different views and orientations. The objects were positioned with random orientation in a complex light field (Debevec, 1998); the viewing position was randomly sampled from a spherical perimeter surrounding the objects (see for details: Toscani et al., 2013a). Reflections were simulated with the physically based rendering system RADIANCE (Ward, 1994) interfaced with a MATLAB toolbox (Heasly et al., 1994). The background was masked by a black and white grid pattern, to standardize local contrasts. Each shape was rendered with the following diffuse reflectance parameters (0.29, 0.35, 0.38, 0.41, 0.44, 0.47, 0.5, 0.53, 0.56, 0.5916, 0.65) and 30 different random orientation and viewing conditions. The central reflectance value (i.e., 0.47) was chosen as reference and the other ones were comparisons, in a constant stimuli paradigm.

### Procedure

In a dark room, participants had their heads stabilized by a chinrest with 58 cm distance between forehead and the center of the screen. They were presented with the reference and comparison stimuli next to each other, randomly left and right or vice versa (Figure 1(a)). The task was to indicate which one was “painted with a lighter gray”. In the *Full* condition, the comparison and the reference were presented as they were rendered, with no manipulation. In the *Dark Cut* condition, the 20% darkest pixels (Figure 1(b), blue area -- left panel) were substituted with the background (Figure 1(b) -- central panel); in the *Light Cut* condition, the 20% lightest pixels (Figure 1(b), red area -- left panel) were substituted (Figure 1(b) -- right panel). For each of the three conditions and each of the two shapes, each reflectance comparison was shown 30 times, at random orientations and viewing angles, for a total of 1980 experimental trials. Presentation order was randomized.

### Analyses

For each condition and each participant, we modeled the probability of the comparison to be reported as lighter than the reference as a function of the reflectance difference. To do that, we used the psignifit 4 (Schütt, Harmeling, Macke, & Wichmann, 2016) MATLAB toolbox to fit a psychometric function to the observers’ responses (with a cumulative Gaussian function). To stabilize the fitting procedure, data were binned into five intervals. The slope of the psychometric function is a measure of JND (just noticeable difference), that is, the albedo difference at which the participants perform discrimination with an 84% success rate. Steeper curves correspond to better ability to tell apart objects with different albedos. We used a one-way repeated measure analysis of variance (ANOVA) on the JNDs to test for overall differences between the three conditions (*Full*, *Light Cut*, and *Dark Cut*) and Bonferroni-corrected *t* tests to assess difference between all condition pairs.

## Results

**Figure 2.**
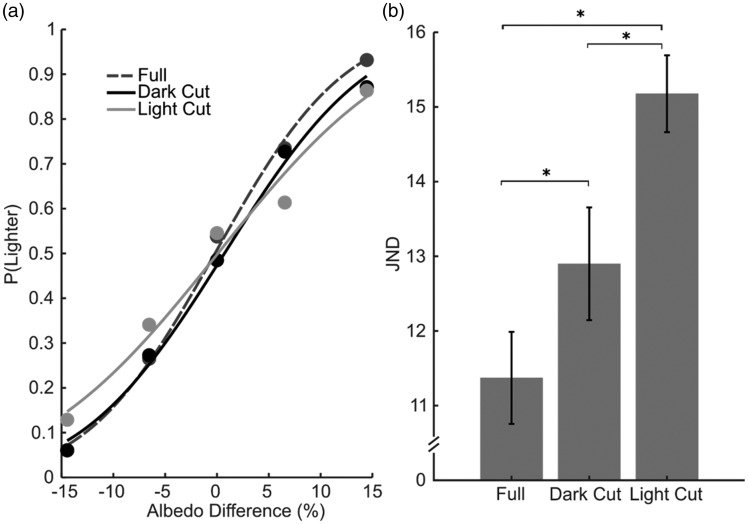
Results. (a) Example of psychometric functions for one observer for the three conditions. *y*-axis denotes the probability of judging the comparisons as lighter than the reference; *x*-axis denotes albedo difference between the comparison and the reference. Albedo is expressed in percentage from black (0%) to white (100%). Black circles indicate the measured probabilities in the *Dark Cut* condition, gray in the *Full* and light gray in the *Light Cut* condition. The black solid line represents the fitted psychometric function for the *Dark Cut* condition, the solid light gray line for the *Light Cut* condition, and the dashed gray line for the *Full* condition. (b) JND averaged across observers (*y*-axis), for the three conditions (*x*-axis). Error bars represent the standard error of the mean. JND = just noticeable difference. (*p < 0.017).

Figure 2(a) shows an example of the psychometric functions for the three conditions of a participant. The slope is steepest in the *Full* condition (dashed gray line), indicating that both replacing the dark and the light portions impaired performance. Crucially, the slope is shallowest in the *Light Cut* condition, suggesting that replacing the light portions of the objects impairs performance the most.

Figure 2(b) represents the average JND in the three conditions. ANOVA reveals a significant difference between conditions, *F*(2,14) = 27.312, *p* < .001.

JNDs are lowest in the *Full* condition, indicating better performed when all the information was available. Both removing the light and dark portions impaired performance—*t*(4) = 4.21 and *t*(4) = 6.12, respectively; *p* values < α, with α = .017 because of the Bonferroni correction. However, the negative effect on performance was bigger in the *Light Cut* than in the *Dark Cut* condition, *t*(4) = 4.25, *p* < .017, indicating the light portions have an higher influence on performance than the dark portions.

## Discussion

We tested how lightness discrimination performance depends on the light and the dark portions of shaded three-dimensional objects. Our results indicate that the light portions are particularly important for lightness discrimination. This is in agreement with our previous results from physically based rendering simulations (Toscani et al., 2013a, 2015; Toscani, Valsecchi, et al., 2017), which could predict perception without incorporating the nonlinear relationship between luminance and perceived brightness (Fechner, 1860; Stevens, 1957). Also cutting the darkest portions impaired performance, suggesting that, although discrimination mostly depends on the lightest portions, the whole distribution is taken into account.

Discrimination and appearance are different perceptual processes characterized by specific but also shared common mechanisms. For instance, color appearance and discrimination are both influenced by a slow color adaptation process, but extremely rapid adaptation exclusively influences appearance (Rinner & Gegenfurtner, 2000).

We argue that lightness appearance of shaded objects is built by selecting the brightest areas of an object’s surface, which are the most informative for lightness discrimination. This strategy supports the discrimination of objects with different albedos and at the same time stabilizes lightness perception when viewing conditions change. In fact, if an object exhibits enough geometrical complexity, some portion of it is surface will be under direct illumination, thus reflecting the largest amount of light possible, given the illuminant and the surface reflectance. This portion would be the brightest one, while other portions would be in shadow.

Using the maximum luminance within the boundaries of an object to estimate lightness is an adaptive strategy because the maximum luminance value is maximally diagnostic, based on the fact that, given a complex enough geometry, one portion of the surface will always be maximally illuminated. This offers an interesting parallel with the problem of estimating the relative lightness of objects within a scene. Again, assuming that there is a complex enough assortment of lightness values within the scene, the highest brightness will correspond to a white or close to white object. Indeed, among the strategies that the visual system uses achieve lightness constancy, one is to assume that the highest luminance in an image is assumed to be white, “anchoring” the perceived albedos of the other surfaces. The lightness of each surface is proportional to its luminance, but dependent on the other surfaces in the scene, being the scale of variability normalized to a fixed range (Gilchrist et al., 1999). These phenomena could be the results of Bayesian integration of prior knowledge on illumination and reflectances in the word with the current sensory evidence (Brainard & Freeman, 1997; Murray, 2013). In general, the visual system seems to estimate the illuminant from the brightest light in the scene (Gilchrist & Soranzo, 2019; Kozaki, 1973; Noguchi & Masuda, 1971), consistent with the “anchoring” rule, since a white surface reflects almost all the light that received from the illumination.

Nevertheless, constancy is far from perfect: When observers are asked to judge lightness, their behavior is often—at last in part—driven by perceived luminance (i.e., brightness; Ripamonti et al., 2004; Robilotto & Zaidi, 2004, 2006; Toscani et al., 2016; Zdravković, 2008). When an object moves through an environment in which the illumination changes across space, its lightness dramatically depends on the intensity of the illumination (Toscani et al., 2016; Zdravković, 2008). Furthermore, some participants systematically base their lightness judgments on albedo, others on brightness (Robilotto & Zaidi, 2004). Thus, lightness and brightness are not trivial to dissociate and it is possible that in the present study, instead of discriminating reflectance differences, observers based their judgments on brightness. However, whether they used a simple (e.g., mean perceived luminance), a more complex brightness measure (e.g., Robilotto & Zaidi, 2004), or measure of perceived surface albedo (i.e., lightness), our results show that in a discrimination task this measure depends more on the brightest than on the darkest portions of the surfaces’ luminance distribution.

Exactly what mechanism implements the heuristic of relying more on the brightest part of an object for recovering albedo is not yet clear. One possibility is that observers only consider the lightest portions of objects for their judgments and ignore the rest. Alternatively, they might compute albedo integrating the perceived luminance distribution, after this has been shifted towards higher values through fixation behavior. In fact, there is evidence that luminance at fixation is extrapolated to peripheral portions of the rest of the surface (Toscani, Gegenfurtner, & Valsecchi, 2017). Both possibilities are consistent with the finding that while judging the lightness of real three-dimensional objects, observers tend to look at their brightest portions and when forced to a different fixation pattern, their lightness judgments change depending on the luminance at fixation (Toscani et al., 2013a, 2013b, 2015, 2016).

Extrapolating perceived luminance based on fixation and fixating the most diagnostic areas within an object could contribute to compress differences within an objects surface and enhancing differences between different objects and their parts, ultimately helping to discriminate different objects. This is similar to what happens for color. Chromatic discrimination, as inferred from the shape of the MacAdam ellipses (MacAdam, 1942), is worse along the direction of elongation of the color distribution of colored natural objects (Ennis, Schiller, Toscani, & Gegenfurtner, 2018; Hansen, Giesel, & Gegenfurtner, 2008). This implies, for instance, humans are poorly sensitive to the different shades of yellow within the surface of a lemon, but can tell apart the color of the lemon from the one of an orange.

The brightest areas within the object surface determine the lightness of matte objects (Toscani, Valsecchi, et al., 2017). This is however not the case for glossy objects, presumably because the visual system ignores specular highlights when judging lightness (Toscani, Valsecchi, et al., 2017). Specular highlights tend to be colocated with the areas that are best illuminated (Koenderink & van Doorn, 1980); therefore, they cover the areas that would be brightest if the object was matte. Based on the results of the present study, ignoring specular highlights should impair lightness discrimination similar to removing the brightest regions of a matte object. Indeed, lightness discrimination is more difficult for glossy surfaces (Toscani, Valsecchi, et al., 2017).

To summarize, object regions which are relied upon for lightness appearance are also more important for lightness discrimination. We argue that the strategy of basing appearance on the most discriminable regions helps to build useful perceptual representations for object recognition, coherent with the idea that the main purpose of lightness and color perception is to recover the properties of objects and materials in our environment, rather than precisely representing the light that reaches our eyes (Witzel & Gegenfurtner, 2018).

## Authors' Note

Matteo Valsecchi is also affiliated with Department of Psychology, Bologna University, Bologna, Italy.

## References

[bibr1-2041669519884335] Adelson E. H. (2000). Lightness perception and lightness illusions In Gazzaniga M. (Ed.), The new cognitive neuroscience (2nd ed., pp. 339–351). Cambridge, MA: MIT Press

[bibr2-2041669519884335] Anderson B. L. (1997). A theory of illusory lightness and transparency in monocular and binocular images: The role of contour junctions. Perception, 26, 419–453. 9404492 10.1068/p260419

[bibr3-2041669519884335] Anderson B. L. (2003). The role of occlusion in the perception of depth, lightness, and opacity. Psychological Review, 110, 785. 14599243 10.1037/0033-295X.110.4.785

[bibr4-2041669519884335] Anderson B. L. Winawer J. (2005). Image segmentation and lightness perception. Nature, 434, 79–83. 15744303 10.1038/nature03271

[bibr5-2041669519884335] Brainard D. H. Freeman W. T. (1997). Bayesian color constancy. Journal of the Optical Society of America A, 14, 1393–1411. 10.1364/josaa.14.001393 9203394

[bibr6-2041669519884335] Cornsweet T. (1970). Vision perception. New York, NY: Academic Press.

[bibr7-2041669519884335] Debevec, P. E. (1998). Rendering synthetic objects into real scenes: Bridging traditional and image-based graphics with global illumination and high dynamic range photography. *Proceedings of SIGGRAPH*, *1998*, 189--198.

[bibr8-2041669519884335] Ennis R. Schiller F. Toscani M. Gegenfurtner K. R. (2018). Hyperspectral database of fruits and vegetables. Journal of the Optical Society of America A, 35, B256–B266. 10.1364/JOSAA.35.00B256 29603941

[bibr9-2041669519884335] Ennis R. Toscani M. Gegenfurtner K. R. (2017). Seeing lightness in the dark. Current Biology, 27, R586–R588. 28633024 10.1016/j.cub.2017.05.008

[bibr10-2041669519884335] Fechner G. T. (1860). Elemente der psychophysik. Leipzig, Germany: Breitkopf and Hartel.

[bibr11-2041669519884335] Fleming R. W. (2014). Visual perception of materials and their properties. Vision Research, 94, 62–75. 24291494 10.1016/j.visres.2013.11.004

[bibr12-2041669519884335] Gilchrist A. L. (1977). Perceived lightness depends on perceived spatial arrangement. Science, 195, 185–187. 831266 10.1126/science.831266

[bibr131-2041669519884335] Gilchrist, A. L., Kossyfidis, C., Bonato, F., Agostini, T., Cataliotti, J., Li, X., … Economou, E. (1999). Ananchoring theory of lightness perception. *Psychological Review*, *106*, 795--834. 10.1037/0033-295x.106.4.795 10560329

[bibr13-2041669519884335] Gilchrist A. L. Soranzo A. (2019). What is the relationship between lightness and perceived illumination? *Journal of Experimental Psychology: Human Perception and Performance*. Advance online publication. doi:10.1037/xhp0000675 10.1037/xhp0000675 31556684

[bibr14-2041669519884335] Hansen T. Giesel M. Gegenfurtner K. R. (2008). Chromatic discrimination of natural objects. Journal of Vision, 8, 1–19. 10.1167/8.1.2 18318605

[bibr15-2041669519884335] Heasly B. S. Cottaris N. P. Lichtman D. P. Xiao B. Brainard D. H. (2014). RenderToolbox3: MATLAB tools that facilitate physically based stimulus rendering for vision research. Journal of Vision, 14, 1–22. 10.1167/14.2.6 PMC3919102 24511145

[bibr49-2041669519884335] Kleiner, M., Brainard, D., & Pelli, D. (2007). What's new in Psychtoolbox-3? *Perception*, *36*, 1–16.

[bibr16-2041669519884335] Knill D. C. Kersten D. (1991). Apparent surface curvature affects lightness perception. Nature, 351, 228–230. 2041568 10.1038/351228a0

[bibr17-2041669519884335] Koenderink J. J. van Doorn A. J. (1980). Photometric invariants related to solid shape. Optica Acta: International Journal of Optics, 27, 981–996.

[bibr18-2041669519884335] Kozaki A. (1973). Perception of lightness and brightness of achromatic surface color and impression of illumination. Japanese Psychological Research 15: 194–203.

[bibr19-2041669519884335] Land E. H. McCann J. J. (1971). Lightness and retinex theory. Journal of the Optical Society of America, 61, 1–11. 5541571 10.1364/josa.61.000001

[bibr21-2041669519884335] MacAdam D. L. (1942). Visual sensitivities to color differences in daylight. Journal of the Optical Society of America, 32, 247–274. 10.1364/josa.39.000808 18142394

[bibr22-2041669519884335] Mach E. (1866). Über die physiologische Wirkung räumlich vertheilter Lichtreize [About the physiological effect of spatially distributed light stimuli]. Sitzungsberichte der Wiener Akademie der Wissenschaften, 54, 3.

[bibr23-2041669519884335] Marr D. (1982). Vision: A computational investigation into human representation and processing of visual information. San Francisco, CA: WH Freeman.

[bibr24-2041669519884335] Milojevic Z. Ennis R. Toscani M. Gegenfurtner K. R. (2018). Categorizing natural color distributions. Vision Research, 151, 18–30. 29555302 10.1016/j.visres.2018.01.008

[bibr25-2041669519884335] Motoyoshi I. Nishida S. Sharan L. Adelson E. H. (2007). Image statistics and the perception of surface qualities. Nature, 447, 206–209. 17443193 10.1038/nature05724

[bibr26-2041669519884335] Murray R. F. (2013, March). Human lightness perception is guided by simple assumptions about reflectance and lighting. In *Human vision and electronic imaging XVIII* (Vol. 8651, pp. 865106-1 to 865106-11). Bellingham, WA: International Society for Optics and Photonics.

[bibr27-2041669519884335] Noguchi K. Masuda N. (1971). Brightness changes in a complex field with changing illumination: A re-examination of Jameson and Hurvich's study of brightness constancy. Japanese Psychological Research, 13, 60–69.

[bibr28-2041669519884335] Pizlo Z. (2001). Perception viewed as an inverse problem. Vision Research, 41, 3145–3161. 11711140 10.1016/s0042-6989(01)00173-0

[bibr29-2041669519884335] Poggio T. Koch C. (1985). III-Posed problems early vision: From computational theory to analogue networks. Proceedings of the Royal Society of London B, 226(1244), 303–323.

[bibr30-2041669519884335] Poggio T. Torre V. Koch C. (1987). Computational vision and regularization theory. In M. A. Fischler & O. Firschein (Eds.), *Readings in computer vision* (pp. 638–643). New York, NY: Elsevier.

[bibr31-2041669519884335] Purves D. Shimpi A. Lotto R. B. (1999). An empirical explanation of the Cornsweet effect. Journal of Neuroscience, 19, 8542–8551. 10493754 10.1523/JNEUROSCI.19-19-08542.1999 PMC6783017

[bibr32-2041669519884335] Rinner O. Gegenfurtner K. R. (2000). Time course of chromatic adaptation for color appearance and discrimination. Vision Research, 40, 1813–1826. 10837828 10.1016/s0042-6989(00)00050-x

[bibr313-2041669519884335] Ripamonti, C., Bloj, M., Hauck, R., Mitha, K., Greenwald, S., Maloney, S. I., & Brainard, D. H. (2004). Measurements of the effect of surface slant on perceived lightness. *Journal of Vision*, *4*, 747--763. 10.1167/4.9.7 15493968

[bibr315-2041669519884335] Robilotto, R., & Zaidi, Q. (2004). Limits of lighness identification for real objects under natural viewing conditions. *Journal of Vision*, *4*, 779--797. 10.1167/4.9.9 15493970

[bibr316-2041669519884335] Robilotto, R., & Zaidi, Q. (2006). Lightness identification of patterned three-dimensional, real objects. *Journal of Vision*, *6*, 18--36. 10.1167/6.1.3 PMC2843147 16489856

[bibr33-2041669519884335] Schütt H. H. Harmeling S. Macke J. H. Wichmann F. A. (2016). Painfree and accurate Bayesian estimation of psychometric functions for (potentially) overdispersed data. Vision Research, 122, 105–123. 27013261 10.1016/j.visres.2016.02.002

[bibr34-2041669519884335] Sharan L. Li Y. Motoyoshi I. Nishida S. Adelson E. H. (2008). Image statistics for surface reflectance perception. Journal of the Optical Society of America A, 25, 846–865. 10.1364/josaa.25.000846 18382484

[bibr35-2041669519884335] Singh V. Cottaris N. P. Heasly B. S. Brainard D. H. Burge J. (2018). Computational luminance constancy from naturalistic images. Journal of Vision, 18, 1–17. 10.1167/18.13.19 PMC6314111 30593061

[bibr36-2041669519884335] Stevens S. S. (1957). On the psychophysical law. Psychological Review, 64, 153. 13441853 10.1037/h0046162

[bibr37-2041669519884335] Thompson W. Fleming R. Creem-Regehr S. Stefanucci J. K. (2011). Visual perception from a computer graphics perspective. Boca Raton, FL: CRC Press.

[bibr38-2041669519884335] Toscani M. Gegenfurtner K. R. Valsecchi M. (2017). Foveal to peripheral extrapolation of brightness within objects. Journal of Vision, 17, 1–14. 10.1167/17.9.14 28837970

[bibr39-2041669519884335] Toscani M. Valsecchi M. Gegenfurtner K. R. (2013a). Optimal sampling of visual information for lightness judgments. Proceedings of the National Academy of Sciences, 110, 11163–11168. 10.1073/pnas.1216954110 PMC3704015 23776251

[bibr40-2041669519884335] Toscani M. Valsecchi M. Gegenfurtner K. R. (2013b). Selection of visual information for lightness judgements by eye movements. Philosophical Transactions of the Royal Society of London B: Biological Sciences, 368, 20130056. 24018718 10.1098/rstb.2013.0056 PMC3758199

[bibr41-2041669519884335] Toscani M. Valsecchi M. Gegenfurtner K. R. (2015). Effect of fixation positions on perception of lightness. *Proceedings of SPIE - The International Society for Optical Engineering,* 9394. doi:10.1117/12.2175673

[bibr42-2041669519884335] Toscani M. Valsecchi M. Gegenfurtner K. R. (2017). Lightness perception for matte and glossy complex shapes. Vision Research, 131, 82–95. 28025053 10.1016/j.visres.2016.12.004

[bibr43-2041669519884335] Toscani M. Zdravković S. Gegenfurtner K. R. (2016). Lightness perception for surfaces moving through different illumination levels. Journal of Vision, 16, 1–18. 10.1167/16.15.21 28006071

[bibr44-2041669519884335] Ward G. J. (1994). The RADIANCE lighting simulation and rendering system. In *Proceeding SIGGRAPH '08* (pp.459–472). New York, NY: ACM Press.

[bibr45-2041669519884335] Wiebel C. B. Toscani M. Gegenfurtner K. R. (2015). Statistical correlates of perceived gloss in natural images. Vision Research, 115, 175–187. 25937518 10.1016/j.visres.2015.04.010

[bibr46-2041669519884335] Witzel C. Gegenfurtner K. R. (2018). Color perception: Objects, constancy, and categories. Annual Review of Vision Science, 4, 475–499. 10.1146/annurev-vision-091517-034231 30004833

[bibr47-2041669519884335] Zdravković S. (2008). Lightness constancy: Object identity and temporal integration. Psihologija, 41, 5–20.

